# Electrodiagnostic Findings in COVID-19 ICU Survivors

**DOI:** 10.7759/cureus.105456

**Published:** 2026-03-18

**Authors:** Daniel A Hu, Benjamin Abramoff, Timothy R Dillingham

**Affiliations:** 1 Physical Medicine and Rehabilitation, University of Pennsylvania, Philadelphia, USA

**Keywords:** covid-19, electrodiagnostic findings, neurophysiology, physical medicine and rehabilitation, sensorimotor polyneuropathy

## Abstract

Background

The long-term effects of COVID-19 on the peripheral nervous system remain incompletely understood. The objective of this study is to characterize electrodiagnostic findings in COVID-19 survivors who required intensive care admissions resulting in persistent weakness and marked functional impairments.

Methodology

A retrospective chart review was conducted on patients who were referred for electrodiagnostic testing between May 2020 and May 2021 and had persistent weakness. This cohort consisted of nine patients: seven males and two females, between 31 and 75 years old.

Results

Nerve conduction studies revealed absent sensory responses in at least one sampled nerve in all patients and absent motor responses in at least one sampled nerve in seven patients. Electromyography in all nine patients demonstrated neuropathic denervation, evidenced by varying degrees of fibrillation potentials and positive sharp waves, as well as long-duration, polyphasic motor units and reduced, neuropathic recruitment. None of the patients had electromyographic evidence of myopathy.

Conclusions

Overall, the electrodiagnostic findings are most consistent with a distal mixed sensorimotor axonal polyneuropathy as the primary pathological process underlying persistent weakness in this cohort. This pattern is consistent with critical illness polyneuropathy and may represent a form of peripheral nerve injury related to severe COVID-19 infection. Further research is needed to better characterize the long-term outcomes and underlying peripheral nerve pathophysiology of COVID-19-associated critical illness polyneuropathy. This may provide important insights into prevention and treatment strategies.

## Introduction

The COVID-19 pandemic has had widespread medical consequences; however, its extrapulmonary manifestations, particularly those affecting the nervous and musculoskeletal systems, are still not well understood. Hospitalized patients with COVID-19 frequently experience complications involving the pulmonary, cardiovascular, and hematologic systems [1]. As a result, many of these patients require extended stays in the intensive care unit (ICU) [2,3]. Critically ill patients are at risk of developing ICU-acquired weakness (ICUAW). ICUAW often presents as limb weakness and/or difficulty weaning from mechanical ventilation. This further leads to prolonged ICU admissions and is associated with increased morbidity, mortality, and chronic functional deficits [4-6].

ICUAW is classified into three subtypes: critical illness myopathy (CIM), critical illness polyneuropathy (CIP), and a combination of both, known as CIM and polyneuropathy (CIMP) [6]. Although the pathophysiology of these neuromuscular conditions is not well known, several risk factors have been identified, including premorbid health status, duration of mechanical ventilation, and the severity of the acute disease [6-8]. The presentation of ICUAW varies depending on the underlying mechanism. CIM is characterized by greater proximal than distal weakness, preserved sensation, and muscle atrophy. In contrast, CIP is characterized by greater distal than proximal weakness, sensory deficits, and limited muscle atrophy [8]. Clinical and electrodiagnostic findings are utilized to distinguish between these entities, which have differing prognoses and recovery trajectories.

Patients diagnosed with COVID-19 may present with symptoms that are consistent with peripheral nervous system pathology [9-12]. These symptoms often include weakness and sensory changes. In some cases, phrenic nerve and diaphragm involvement can lead to difficulty weaning from mechanical ventilation [13]. These symptoms often present during the acute phase of COVID-19, as in ICUAW, and can persist into the sub-acute phases (3-12 weeks post-diagnosis) as well as the chronic phase (more than 12 weeks post-diagnosis) [14].

The objective of this study is to characterize electrodiagnostic findings in COVID-19 survivors who required intensive care. We report electromyography (EMG) and nerve conduction study (NCS) findings from nine patients who were admitted to acute rehabilitation following a COVID-19 diagnosis with persistent weakness and marked functional impairments.

This article was previously presented as a meeting abstract at the 2025 AANEM Annual Scientific Meeting, held October 29-November 1, 2025.

## Materials and methods

Study design

Electronic medical records at one tertiary hospital center were retrospectively searched from May 2020 to May 2021 to identify patients with a COVID-19 diagnosis who were hospitalized in the ICU with neuropathic symptoms and subsequently underwent electrodiagnostic (EDx) evaluation to assess the cause of motor weakness and functional impairments. This study was found to be exempt by the institutional review board.

Inclusion criteria

To be included in the study, patients needed to have a COVID-19 diagnosis requiring ICU stay and neuropathic symptoms, including weakness and paresthesia, that were subsequently evaluated with EDx testing. Patients needed to have been evaluated with both a nerve conduction study (NCS) and electromyography (EMG) as part of their EDx testing to be included.

Exclusion criteria

Patients with neuropathic symptoms who did not have both a COVID-19 diagnosis and a stay in the ICU were excluded from our study. Patients were also excluded if they did not undergo EDx testing.

Data collection

Data were collected by one reviewer through retrospective review of patient charts and included patient demographics, details of acute care hospital course, and EDx findings. EDx findings are composed of NCS responses and EMG results, including the presence of spontaneous muscle activity, signs of denervation, and motor unit recruitment patterns.

Electrodiagnostic protocol

The Edx protocol consisted of a detailed physical examination and sensory and motor nerve conduction studies, and concentric needle electromyography of weak and non-involved muscles selected based on the patient’s clinical presentation and differential diagnosis. All electrodiagnostic tests were completed with Cadwell electrodiagnostic instruments. Patients’ verbal consents were obtained before beginning the procedure, with the patient able to stop the procedure at any time. Standard electrodiagnostic procedures were conducted.

## Results

Patient characteristics

Nine patients with confirmed COVID-19 and clinical features suggestive of peripheral nervous system dysfunction, who underwent electrodiagnostic testing, were included. Demographic data, clinical characteristics, and EDx findings are summarized in Tables 1-2.

**Table 1:** Patient demographics and clinical features. TIIDM, type II diabetes mellitus; CVA, cerebrovascular accident; TFESI, transforaminal epidural steroid injections; CRRT, continuous renal replacement therapy; DVT, deep vein thrombosis; AKI, acute kidney injury; HD, hemodialysis; BLE, bilateral lower extremities; SAH, subarachnoid hemorrhage; PE, pulmonary emboli; XRT, radiation therapy

Patient	Age (years)	Sex	TIIDM diagnosis	Pertinent other co-morbidities	Duration of acute hospitalization (days)	Pertinent complications of hospitalization	Four extremity weakness	Presence of sensory changes
1	31	M	No	None	122	Septic shock, AKI requiring HD, encephalitis	Yes	Yes
2	38	M	No	Hyperthyroidism	60	VV ECMO	Yes	No
3	69	F	Yes	None	70	SAH, PE, AKI requiring CRRT	Yes	Yes
4	60	M	Yes	Previous XRT, CVA	41	Acute cerebral infarct	Yes	No
5	58	M	Yes	None	112	Septic shock	Yes	Yes
6	74	M	No	None	167	DVT in the BLE	Yes	No
7	67	M	No	None	68	None	Yes	Yes
8	68	M	No	None	73	None	Yes	Yes
9	63	F	Yes	CVA	71	AKI requiring CRRT	Yes	Yes

**Table 2:** Summary of NCS/EMG findings. *Patient did not tolerate EMG, and only one muscle was sampled. A, absent; D, diminished; N, normal; Tib Ant, tibialis anterior; EHL, extensor hallucis longus; MGast, medial gastrocnemius; Ext Dig, extensor digitorum; FDI, first dorsal interossei; APB, abductor pollicis brevis; RF, rectus femoris; VastM, vastus medialis; BB, biceps brachii; NCS, nerve conduction study; EMG, electromyography

Patient	EMG denervation	Fibrillation potentials (degree)	Positive sharp waves (degree)	Absent sensory response	Nerves sampled	Absent motor response	Nerves sampled	Motor unit recruitment pattern	Final diagnosis
1	Yes*	Right Tib Ant (3+)	Right Tib Ant (3+)	Yes	Right Median (D); Right Superficial Peroneal (N); Right Sural (A)	Yes	Left Median (N); Right Peroneal (A)	Neuropathic	Polyneuropathy
2	Yes	Right EHL (1+); Right MGast (1+); Left EHL (1+)	Right EHL (2+); Right MGast (1+); Left EHL (2+)	Yes	Right Median (D); Right Sural (A)	No	Right Median (N); Right Peroneal (N)	Neuropathic	Polyneuropathy
3	Yes	Right Tib Ant (3+); Left MGast (3+)	Right Tib Ant (3+); Left MGast (3+)	Yes	Right Median (A); Right Superficial Peroneal (A)	Yes	Right Median (N); Right Peroneal (A)	Neuropathic	Polyneuropathy
4	Yes	Right Tib Ant (2+; Right MGast (2+); Right Ext Dig (3+); Right FDI (3+) Left MGast (2+); Left Tib Ant (2+)	Right Tib Ant (3+); Right MGast (1+); Right Ext Dig (2+); Right FDI (2+); Left MGast (2+); Left Tib Ant (2+)	Yes	Right Radial (A); Right superficial peroneal (A)	No	Right Ulnar (D); Right Peroneal (N)	Neuropathic	Polyneuropathy
5	Yes	Right Tib Ant (1+); Right MGast (1+); Left Tib Ant (4+); Left MGast (4+); Left FDI (1+)	Right Tib Ant(2+); Right MGast (2+); Left Tib Ant(4+); Left MGast (4+); Left FDI (1+)	Yes	Left Radial (A); Right Sural (N)	Yes	Right Peroneal (A); Left tibial (A)	Neuropathic	Polyneuropathy
6	Yes	Right FDI (4+); Right Ext Dig (4+); right APB (3+); Left FDI (2+); Left Tib Ant (1+)	Right FDI(4+); Right Ext Dig (4+); Right APB (2+); Left FDI (2+); Left Tib Ant (1+)	Yes	Right Radial (A); Right Median (D); Left Sural (A)	Yes	Right Median (N); Left Peroneal (A)	Neuropathic	Polyneuropathy
7	Yes	Right Tib Ant (2+); Right MGast (1+); Right RF (1+); left Tib Ant (2+)	Right FDI(1+); Right Tib Ant (2+); Right MGast (2+); right RF (2+); left Tib Ant (2+)	Yes	Right Median (D)' Right Sural (A); Right Ulnar (A)	Yes	Right Median (N); Right Peroneal (A); Right Ulnar (N)	Neuropathic	Polyneuropathy
8	Yes	Right FDI (1+); Right EHL (2+); Left Tib Ant (3+)	Right FDI (1+); Right EHL (2+); Left Tib Ant (3+)	Yes	Right Median (D); Right Sural (A)	Yes	Right Median (N); Right Peroneal (A)	Neuropathic	Polyneuropathy
9	Yes	Right Tib Ant (2+); Right MGast (1+); Right VastM (2+); Right FDI (1+); Right BB (3+); Left MGast (2+); Left VastM (1+); Left BB (2+)	Right Tib Ant (2+); Right MGast (2+); Right VastM (2+); Right FDI (1+); Right BB (2+); Left MGast (2+); Left VastM (1+); Left BB (2+)	Yes	Right Radial (N); Right Sural (A)	Yes	Right Peroneal (A); Left Tibial (A)	Neuropathic	Polyneuropathy

The cohort consisted of seven males and two females, with an average age of 58.7 years. All patients required ICU-level care for ventilator support during their acute illness. Acute hospital stays were prolonged, with an average length of stay of 87.1 days. Upon admission to acute rehabilitation, all patients had generalized weakness affecting all four extremities. Six patients also presented with sensory deficits. Five patients had pertinent comorbidities that have been shown to contribute to peripheral nervous system dysfunction (Table 1). None of the patients had undergone EDx testing before their rehabilitation admission.

Nerve conduction studies

All patients had absent sensory responses in at least one nerve sampled. Seven out of nine patients had absent motor responses in at least one nerve. Of the two patients with preserved motor responses, one patient had normal responses in the nerves sampled, and one patient had a diminished response in one nerve sampled (Table 2).

Electromyography

**Figure 1:**
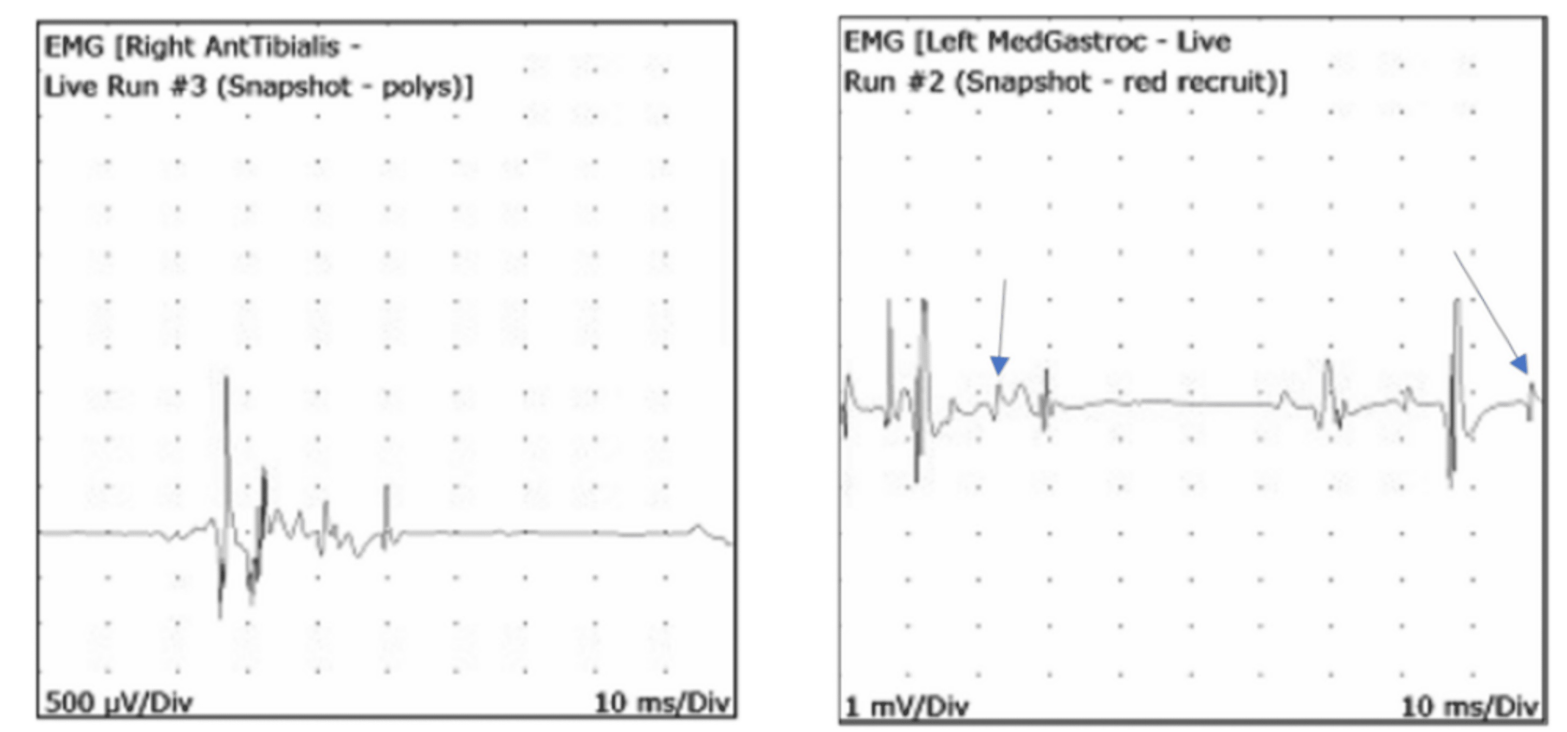
Waveform examples from two separate patients, both representing long-duration polyphasics, with the right also demonstrating reduced recruitment and satellite potentials (arrows), consistent with and signifying neuropathic and ongoing reinnervation in the muscles tested.

EMG findings in all nine patients demonstrated evidence of denervation, characterized by varying degrees of fibrillation potentials and positive sharp waves (Table 2). One study was limited to one muscle due to limited patient tolerance. Motor unit action potentials on concentric needle EMG demonstrated long-duration polyphasic potentials with reduced neuropathic recruitment consistent with neuropathic motor unit dysfunction and ongoing motor unit reinnervation (Figure 1). There were no cases in which myopathic potentials were seen, nor was there any evidence of early (myopathic) recruitment. Findings in all muscles were reflective of neuropathic dysfunction.

The electrodiagnostic conclusions in all nine patients were sensory and motor axonal polyneuropathy.

## Discussion

CIP is well documented as a potential sequel of prolonged hospital stays and ICU admissions. Known risk factors include illness severity, duration of ICU admission, and comorbidities, including sepsis, systemic inflammatory response syndrome, multi-organ failure, female sex, severe asthma, electrolyte abnormalities, malnutrition, immobility, central neurologic failure, renal failure, renal replacement therapy, and vasopressor support [15,16]. Here, we describe the development of CIP in nine patients with a COVID-19 diagnosis who required mechanical ventilation. These patients had varying lengths of stay, comorbidities, and complications similar to findings observed in critically ill patients without COVID-19 who developed CIP.

Although the literature is limited, existing literature has utilized EDx studies to explore the association between COVID-19 and neuromuscular weakness. Bagnato et al. analyzed EDx findings in 21 patients admitted to rehabilitation following COVID-19, identifying pathologic neuromuscular findings in 17. Among these, five were diagnosed with CIMP, five with CIP, four with CIM, two with Guillain-Barré Syndrome, and two with other nerve pathologies [17]. Similarly, Cabañes-Martínez et al. studied 12 patients with suspected CIP or CIM following COVID-19, finding four patients with sensory-motor axonal polyneuropathy and seven patients with evidence of myopathy [6]. Another case report by Daia et al. examined three patients with COVID-19 presenting with lower extremity myalgias who did not require ICU admission and found their EDx findings were most consistent with a demyelinating polyneuropathy [18]. Additionally, a scoping review by Intiso et al. compiled information from 80 patients across 11 studies on ICUAW following COVID-19 and found CIM in 23 patients, CIP in 21 patients, and CIMP in 15 patients [19].

More recent, retrospective cross-sectional studies by Torres-Carranza et al. demonstrate nerve abnormalities in 64.11% of 170 patients, including conduction velocity, peak latency, and amplitude impairments [20]. Khalil et al. similarly showed that of the 55 patients examined, polyneuropathy was observed in 40% of patients and was the most common diagnosis [21]. While our study focused on patients with COVID-19 who had an ICU stay, weakness and sensory symptoms have also been reported in individuals with long COVID. In a study by Oaklander et al., 59% of 17 patients with sensory symptoms and long COVID had evidence of neuropathy, including critical illness polyneuropathy, multifocal demyelinating neuropathy, and small fiber neuropathy [22]. These studies were consistent with the findings of our study, which also demonstrated a distal mixed sensorimotor polyneuropathy associated with COVID-19 in our patients. We did not find evidence of demyelination in our subjects. All of the present cohort consisted of patients with CIP. We did not see a distribution of diagnoses that Intiso et al. reported [19]. Given the rise in human population, climate change, and increased globalization, future pandemics like COVID-19 are increasingly likely [23]. In these electrodiagnostic scenarios, our findings may be useful to inform care by providing insight into the relationship between illness and neuromuscular dysfunction. The underlying neuropathophysiology should focus on the peripheral nerve causes.

One potential reason that other researchers might have found some evidence of myopathy is that they identified satellite potentials that looked like myopathic potentials. On our Cadwell instrumentation, we record an epoch of free-running needle EMG recordings, then we can freeze this selected time period and go back through the motor units. It was impressive the underlying reinnervation that was taking place with long-duration polyphasic potentials with many satellite potentials that had the appearance of myopathic potentials if only observed in free-running EMG mode. In our EDx evaluations, we have the patient conduct a submaximal lower-level recruitment so that the motor unit action potentials are analyzable.

This study is not without limitations. The small sample size of patients from one institution who are critically ill with COVID-19 limits generalizability to the broader population who may be affected by COVID-19. Although clinical context supports CIP as a cause of neuropathy, comorbidities in some patients, including hypothyroidism, renal disease, and diabetes, are confounding factors. Another limitation is the lack of data on prognosis and recovery following neuromuscular injury due to COVID-19. Future research involving a larger and more diverse cohort of patients with COVID-19 across multiple institutions, both with and without ICU-level care requirements, is necessary to further delineate the peripheral nerve dysfunction associated with COVID-19 and better understand the prognosis and recovery following such injuries.

## Conclusions

EDx findings from our study suggest that a distal sensorimotor axonal polyneuropathy can be found in patients with COVID-19 who required ICU-level care. All patients in our study demonstrated neuropathic motor unit recruitment patterns, suggesting axonal loss rather than myopathy. Several patients also demonstrated significant satellite and polyphasic potentials, indicating active reinnervation of the muscles tested. Future studies including serial EDx evaluations allow for longer follow-up, which would provide valuable information regarding long-term prognosis and recovery timeline in ICU patients with COVID-19 and neuropathic symptoms. Additionally, further research, including basic science, translational, and clinical studies, is needed to explore the underlying peripheral nerve pathophysiology that could elucidate insights into prevention and treatment strategies.
